# Handlebar Robotic System for Bimanual Motor Control and Learning Research

**DOI:** 10.3390/s21185991

**Published:** 2021-09-07

**Authors:** Lucas R. L. Cardoso, Leonardo M. Pedro, Arturo Forner-Cordero

**Affiliations:** 1 Programa de Pós-Graduação Interunidades em Bioengenharia (EESC/FMRP/IQSC), University of São Paulo, São Carlos 13566-590, Brazil; 2 Department of Mechanical Engineering, Federal University of São Carlos, São Carlos 13565-905, Brazil; lmpedro@ufscar.br; 3 Biomechatronics Laboratory—Escola Politecnica, University of São Paulo, São Paulo 05508-010, Brazil; aforner@usp.br

**Keywords:** bimanual coordination, motor learning, motor control, robotic handlebar, robotic training

## Abstract

Robotic devices can be used for motor control and learning research. In this work, we present the construction, modeling and experimental validation of a bimanual robotic device. We tested some hypotheses that may help to better understand the motor learning processes involved in the interlimb coordination function. The system emulates a bicycle handlebar with rotational motion, thus requiring bilateral upper limb control and a coordinated sequence of joint sub-movements. The robotic handlebar is compact and portable and can register in a fast rate both position and forces independently from arms, including prehension forces. An impedance control system was implemented in order to promote a safer environment for human interaction and the system is able to generate force fields, suitable for implementing motor learning paradigms. The novelty of the system is the decoupling of prehension and manipulation forces of each hand, thus paving the way for the investigation of hand dominance function in a bimanual task. Experiments were conducted with ten healthy subjects, kinematic and dynamic variables were measured during a rotational set of movements. Statistical analyses showed that movement velocity decreased with practice along with an increase in reaction time. This suggests an increase of the task planning time. Prehension force decreased with practice. However, an unexpected result was that the dominant hand did not lead the bimanual task, but helped to correct the movement, suggesting different roles for each hand during a cooperative bimanual task.

## 1. Introduction

Motor control and motor learning are two broad and interrelated concepts that encompass the ability to acquire and execute skilled motor performance despite unknown dynamics or unpredictable perturbations. More precisely, motor learning can be considered as an update of the internal model of the task [1] that results in a performance improvement, a skill memory (retention over longer time), and transfer of the skill to similar tasks performed under different conditions. The comprehension of the underlying processes behind both concepts has a wide range of practical relevance, from physical therapists to athletes, both connected to the idea that a better performance depends on practice [2].

Research on this field is supported by experimental paradigms that aim at exploring the three major components of a motor skill: movement goal formation, action selection to achieve the goal and the execution itself [2,3,4,5]. Some examples are the force field paradigm [6,7], used to study motor adaptation; the stimulus–response paradigms [5,8,9], associated to both action selection and execution of the motion; and the motor acuity paradigms [10,11,12], exclusively related to action execution. Instrumented and actuated devices, such as robots, are also employed to expand the scope of these experiments, recording kinematic (position, speed) and dynamic (force, torque) data, as well as providing perturbing forces or sensory feedback (visual, haptic or sounds) [5,6,7,8,9,10,11,12].

Most of the motor control and learning studies with robots were designed for unilateral tasks, elucidating little information about the mechanisms involved in bilateral coordination or dexterity. This is particularly disadvantageous in the context of rehabilitation. Improvements in functionality after a neurological disease (e.g., stroke) rely on motor training and practice-induced plasticity [13], thus the neural processes associated to the positive outcomes can be described as a type of motor learning [14,15]. However, diminished bimanual motor performance observed in hemiparetic patients cannot be attributed only to the affected limb, but also results from a bilateral coordination deficit. Some studies showed that bilateral training engages specific cortical areas [16,17,18]. This means that the coordinated control of both limbs is not equivalent to the superposition of controlling each limb separately [19]. Moreover, results presented in the literature suggest a limited transfer from unimanual to bimanual functions [20]; conversely, others confirmed the transfer in the opposite way [21].

The understanding about motor control and learning in unimanual tasks has been successfully translated from the research laboratories to the clinical settings [13,22]. On the other hand, functional outcomes obtained through bimanual training devices designed for neuro-rehabilitation [23,24,25] are still controversial [19,26,27]. Different literature reviews reported that the data supporting the clinical benefits of using bilateral training devices were inconclusive [28,29], and a recent review about the subject still suggests restoring interlimb coordination function [30].

These inconsistent results between robotic assistance in unimanual or bimanual conditions may be connected to the need for studies that elaborate on the mechanisms behind the bilateral training interventions, including the hand dominance, before working on brand new devices for clinical rehabilitation. Addressing this issue, some authors have implemented motor learning paradigms in bimanual environment, using robotic systems to study adaptation due to force field [21,31,32,33], proposing bimanual versions of the serial reaction time task [34,35,36,37], or investigating motor acuity by analysing the prehension force during bimanual manipulation [38,39].

In line with these studies, the present work shows the design, construction and validation of a new device designed to implement different motor learning paradigms for bimanual motor learning research. Experimental validation was performed with healthy subjects, using reaction time and timed-response tasks. In addition, the apparatus can implement force field and motor acuity paradigms. Using six separate force sensors and a rotational encoder, the system combines kinematic data (e.g., acceleration) with dynamic measurements (including grip strength) from each upper limb. This information can be used to explain the processes and mechanisms involved in bimanual control in healthy or disabled people.

Force recordings during bimanual manipulation are important to quantitatively analyse the coordination control among limbs, in stroke survivors [40,41,42] or healthy and elderly adults [39,43,44,45,46]. Additionally, a recent literature review with meta-analysis showed that patients with stroke have dynamic imparement control of bimanual coordination [30]. Up to date, to our knowledge, previous bimanual robotic devices cannot measure simultaneously coupled arm manipulation and grip force independently for each arm.

Based on prior studies with unimanual manipulation and the current knowledge about bimanual force control, we tested two motor learning hypotheses with our handlebar robot: (1) From a kinematic perspective, if the task is sufficiently challenging, (i) movement accuracy should increase with practice whereas the (ii) reaction time and (iii) movement velocity should decrease. (2) From a dynamic perspective, (i) prehension force should decrease with practice, but only when kinematic adaptation is observed, (ii) the prehension force of the dominant hand should always be stronger than that of the non-dominant hand, (iii) the dominant hand should tend to lead the task and (iv) cooperation between limbs would tend to increase with practice following the improvement in spatial accuracy.

The paper is organized as follows: Section 2 presents the hardware, the control architecture and the mathematical model. Section 3 describes the experimental protocol and the methodology for the data analysis. Section 4 and Section 5 present and discuss the results, respectively. The conclusions as well as proposals for future research are found in Section 6.

## 2. The Handlebar

**Figure 1:**
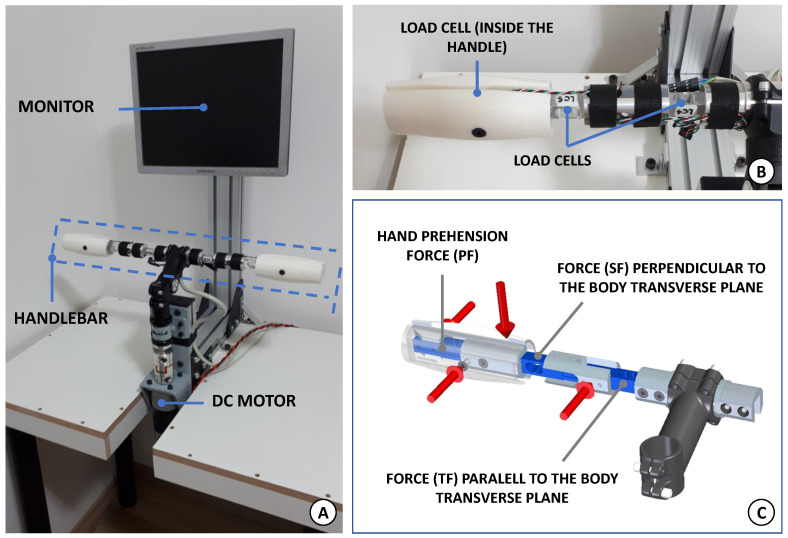
The developed handlebar-like system. (**A**) The instrumented handlebar, DC motor and the 15" monitor are supported with a frame of aluminium profile. (**B**) Left side of the handlebar showing three load cells, mounted in different positions and orientations. (**C**) Each load cell has a specific mount position and orientation to record the different forces.

In order to study the motor learning of coupled and cooperative movements with both limbs, a handlebar-like system, of 1 actuated DOF, was designed and constructed (Figure 1A).

### 2.1. Mechanical and Electronic Design

The control of a handlebar is particularly interesting once it requires a rotational movement, involving the entire upper limb system in a coordinated and specific sequence of joint sub-movements, constantly adjusted by feedback.

As shown in Figure 1B, the proposed handlebar is composed of six load cells (capacity of 5 kg·f each), in order to record the force data during the subject’s interaction. Figure 1C illustrates the forces measured by each load cell (right side), with their specific orientation and position. First, both handles have an inset load cell able to record the user’s hand prehension force (PF). Additionally, other load cells are installed so as to measure the subject’s force that is perpendicular to the user’s body transverse plane. This measurement can be associated to the subject’s ability of supporting their own arm, the deficit of which, among stroke survivors, for example, may lead to shoulder subluxation. Due to this characteristic, hereinafter this force will be denoted as supporting force (or SF). Finally, there is a third load cell on each side, responsible for monitoring the applied forces parallel to the body transverse plane (indicated by TF), which are the ones that mostly contribute to the handlebar rotation.

The rotational axis is coupled with a DC motor with a planetary gearbox (one stage, 1:6) resulting in a stall torque of 17 N·m (at 12 V/30 A) and with an attached quadrature optical encoder of high precision (72,000 counts per revolution). A permanent magnet DC motor was selected due to its linear dynamic behaviour suitable for impedance control implementation. The entire apparatus is mounted on a wood desk and supported with a frame of aluminium profile that also holds a $15^{\prime \prime }$ monitor for the visual interaction.

The load cells signal amplification and offset adjustment are performed using a dedicated microchip (INA125P, Texas Instruments^®^, Dallas, TX, USA), whereas for the decoder and count of the quadrature encoder, the HCTL-2022 is used (Avago Technologies^®^, San Jose, CA, USA) at 8 MHz clock operation, both mounted on an exclusively designed PCB. The DC motor is driven via a high-power H-bridge module, controlled by PWM generated through an Arduino M0 PRO. The Arduino also converts the load cell signals from analog to digital (13-bit resolution), closes the loop for the control system and sends all the data via USB communication, at 530 Hz.

**Figure 2:**
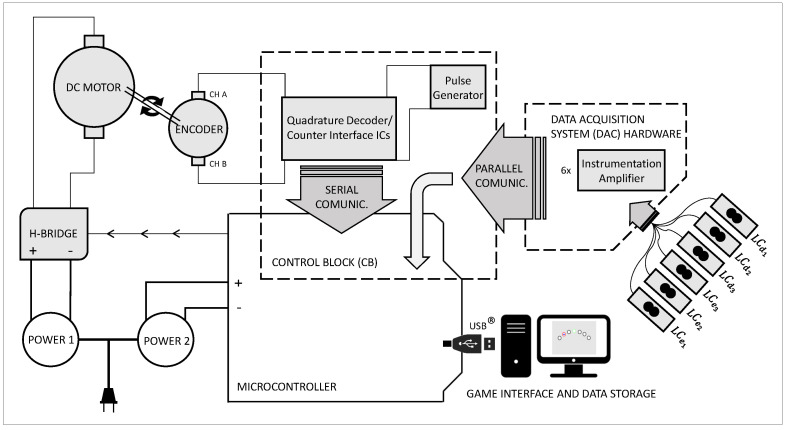
Schematic diagram of the system hardware. The Data Acquisition System includes the instrumentation amplifiers and the loadcells (LC), while the Control Block comprises the ICs for the decoder and count of the quadrature encoder. In order to avoid electrical interference, different power sources were used for the motor and for the electronics.

Figure 2 presents a schematic diagram of the full hardware.

A script in MATLAB^®^ (Natick, MA, USA) was developed for a visual interface and to store the data.

### 2.2. Control Architecture

An impedance control system was chosen to promote a safer environment for the human interaction. It was inspired by [47] and implemented as a position controller, in which the desired position is given so as to respect the dynamics of a mass-spring-damper system. Thus, for the handlebar shown in Figure 1, its angular position (θ(t)), speed ($\dot{\theta }$) and acceleration ($\ddot{\theta }$) need to comply with the following control law

(1) $$T_{m}=I\ddot{\theta }+B\dot{\theta }+K\left(\theta_{0}-\theta \left(t\right)\right)$$

where *I*, *B* and *K* are, respectively, the moment of inertia, the viscous damping, and the stiffness of the joint according to the desired impedance; $\theta_{0}$ is the initial angular position; and $T_{m}$ is the torque measured between the human $T_{h}$ and the DC motor actuator ($T_{act}$), given by

(2) $$T_{m}=T_{h}-T_{act}$$

In a discrete system, the angular speed and acceleration (${\dot{\theta }}_{i}$ and ${\ddot{\theta }}_{i}$) can be described by

(3) $$\left\{\begin{array}{c} {\dot{\theta }}_{i}=\frac{\theta_{i}-\theta_{i-1}}{\Delta t_{i-1}}\\ {\ddot{\theta }}_{i}=\left[\left(\frac{\theta_{i}-\theta_{i-1}}{\Delta t_{i-1}^{2}}\right)-\left(\frac{\theta_{i-1}-\theta_{i-2}}{\Delta t_{i-1}\cdot \Delta t_{i-2}}\right)\right] \end{array}\right.$$

where $\Delta t_{i-1}$ and $\Delta t_{i-2}$ are the sample times of each interaction; however, for simplification, one can assume $\Delta t_{i-1}\approx \Delta t_{i-2}=\Delta t$. After applying simple algebra, the desired position ($\theta_{i}^{d}$) becomes

(4) $$\theta_{i}^{d}=\frac{T_{m}+\theta_{i-1}^{d}\left(\frac{2I}{\Delta t^{2}}+\frac{B}{\Delta t}\right)-\theta_{i-2}^{d}\left(\frac{I}{\Delta t^{2}}\right)-\theta_{0}^{d}K}{\left(\frac{I}{\Delta t^{2}}+\frac{B}{\Delta t}-K\right)}$$

The system was designed to adjust to the desired position via a proportional-derivative (PD) controller, at every cycle. Thus, the resultant torque ($T_{res}$) produced by the sum of both human and motor torques actuates on the handlebar, with an assumed behaviour of a mass-damping system

(5) $$T_{res}=I_{H}\ddot{\theta }+B_{H}\dot{\theta }$$

where $I_{H}$ and $B_{H}$ are, respectively, the moment of inertia and the viscous damping inherent to the system comprising the handlebar and its axis, the coupling and the bearing housing.

**Figure 3:**
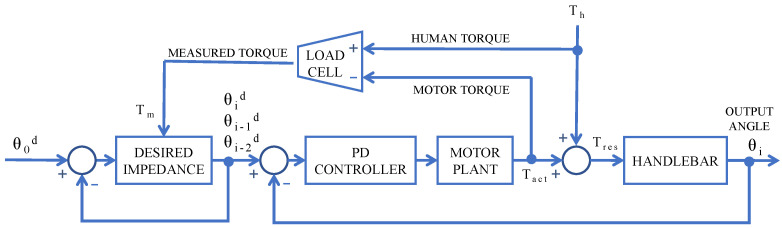
Block diagram of the control algorithm. The Desired Impedance block feeds the PD controller with a position reference, based on (4); thus, the motor and human torque actuate on the handlebar.

The block diagram is presented in Figure 3.

This alternative way of implementing impedance control is different from the one used by [47], but is especially suitable in situations when (i) the motor plant is not totally well established and (ii) the motor is not controlled directly by current, which means that the torque is only indirectly imposed. In the system described here, both characteristics are true, since the motor parameters were not given by the manufacturer and PWM (voltage) is used as the control variable.

### 2.3. Mathematical Model

Due to the design of the handlebar, part of its load cells are simultaneously subjected to more than one force applied by the user during the interaction. Thus, in order to assess the modulus of each interaction force individually, a model is necessary for mechanical decoupling. Additionally, this model is crucial to convert the load cell signals into force measurements with the right unit.

**Figure 4:**
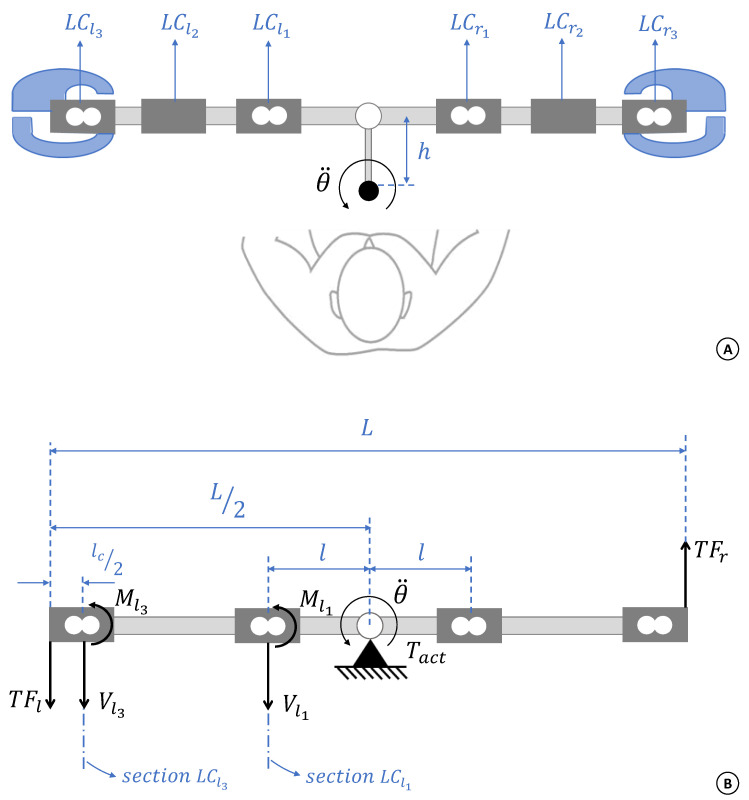
(**A**) Schematic illustration of the handlebar in a top view section. The load cells inside the handles were mounted in a specific way that generates different signals when pushing or pulling the handlebar. (**B**) Free body diagram of the handlebar, showing the two virtual sections used to build the mechanical model of the system.

Figure 4A shows a schematic top view of the handlebar that helps to visualise this coupling issue.

The $LC_{l_{1}}$ (or, symmetrically, the $LC_{r_{1}}$) always captures the movements of the subject’s both arms. The same applies for the $LC_{l_{3}}$ (or $LC_{r_{3}}$) but, in this case, it is also sensitive to the prehension force of the respective hand. Additionally, a more detailed analysis of the handles in Figure 4A shows that the signals of their inset load cells depend on the way the user interacts with the handlebar—pushing or pulling it. In turn, the $LC_{l_{2}}$ (and $LC_{r_{2}}$) are the simplest case, in which they are uniquely exposed to the supporting force of the respective arm (right or left), and a straightforward lever rule can reveal their magnitude.

In order to simplify the model, a constant linear mass density (*d*) was adopted for the handlebar, with mass, *m*, and total length, *L*. The distance *h*, indicated in the Figure 4A, was neglected, assuming the handlebar turns around its geometrical centre.

#### 2.3.1. Forces ${\mathrm{TF}}_{r}$ and ${\mathrm{TF}}_{l}$

According to Figure 1C, ${\mathrm{TF}}_{r}$ and ${\mathrm{TF}}_{l}$ are the user’s right and left arm forces, parallel to the body transverse plane. Considering the representation in Figure 4B, and disregarding the axis friction and dumping coefficients, the dynamic equation for the system can be written as

(6) $$T_{h}+T_{act}=I_{H}\ddot{\theta }$$

where $T_{h}={\mathrm{TF}}_{r}+{\mathrm{TF}}_{l}$ is the resultant torque applied by the user, $T_{act}$ is the torque produced by the actuator, $\ddot{\theta }$ is the angular acceleration and $I_{H}$ is the handlebar moment of inertia, which can be obtained by considering it as a rigid and thin rod with constant linear mass density (*d*).

A virtual section going through the middle of the $LC_{l_{1}}$ (Figure 4B) reveals two additional equations, based on the sum of shear forces, $V_{l_{1}}$, and bending moments, $M_{l_{1}}$ in this section.

Finally, the electrical signal produced by the load cell is a function of the same $V_{l_{1}}$ and $M_{l_{1}}$, as follows:

(7) $$-V_{l_{1}}+\frac{M_{l_{1}}}{\frac{l_{c}}{2}}=\Delta LC_{l_{1}}$$

where symbol Δ denotes the electrical signal produced by the respective load cell and $l_{c}$ is the load cell total length.

Using physical features of the system (Table 1), the matrix below is used to establishthe desired forces, ${\mathrm{TF}}_{r}$ and ${\mathrm{TF}}_{l}$.

(8) $$\begin{array}{c} \left[\begin{array}{cccc} 0.2775 & 0.2775 & 0 & 0\\ 0.2775 & 0 & 0.12 & 1\\ 1 & 0 & 1 & 0\\ 0 & 0 & -1 & 25 \end{array}\right]\cdot \left[\begin{array}{c} {\mathrm{TF}}_{l}\\ {\mathrm{TF}}_{r}\\ V_{x_{3}}\\ M_{x_{3}} \end{array}\right]=\left[\begin{array}{c} 0.0257\cdot \ddot{\theta }-T_{act}\\ 0.0118\cdot \ddot{\theta }-T_{act}\\ -0.0564\cdot \ddot{\theta }\\ \Delta LC_{x_{1}} \end{array}\right] \end{array}$$

where x=l or x=r means that it is possible to use the load cell signals $\Delta LC_{l_{1}}$ or $\Delta LC_{r_{1}}$ to obtain the same force magnitude values. Indeed, for a more accurate result, it is convenient to apply both methods and average out the results.

#### 2.3.2. Forces ${\mathrm{PF}}_{r}$ and ${\mathrm{PF}}_{l}$

The prehension forces of the user’s right and left hands can be obtained through the signals $\Delta LC_{r_{3}}$ and $\Delta LC_{l_{3}}$, respectively. However, this depends on the way the user interacts with the handlebar. For example, let us imagine that the user is rotating the handlebar of Figure 4A in a counter-clockwise direction. One possible way to do that is by pulling the left hand and pushing the right one. In this scenario, the electrical signal $\Delta LC_{r_{3}}$ is directly proportional to the right hand prehension force (${\mathrm{PF}}_{r}$) because the action of pushing the right handle has no effect on its inset load cell. Thus (for both handles), when pulling,

(9) $$\left\{\begin{array}{c} {\mathrm{PF}}_{l}=\Delta LC_{l_{3}}\\ {\mathrm{PF}}_{r}=\Delta LC_{r_{3}} \end{array}\right.$$

It is possible to produce the same rotation by pulling the right hand and also pulling the left one, but with a lower force in the former. In this situation, the electrical signal $\Delta LC_{r_{3}}$ is not sensitive only to the right hand prehension but also to the dynamics produced by the left hand pulling force. Therefore, to find out the magnitude of each prehension force, it is necessary to use a model, similar to matrix (8). It can be obtained following the same steps as described before, but focusing on virtual section $LC_{l_{3}}$. Thus,

(10) $$\begin{array}{c} \left[\begin{array}{ccc} 0.2375 & 1 & 0\\ 1 & 0 & 0\\ -1 & 25 & 1 \end{array}\right]\cdot \left[\begin{array}{c} V_{x_{3}}\\ M_{x_{3}}\\ {\mathrm{PF}}_{x} \end{array}\right]=\left[\begin{array}{c} 0.0048\cdot \ddot{\theta }-T_{act}-0.2775\cdot {\mathrm{TF}}_{x}\\ 0.0186\cdot \ddot{\theta }-{\mathrm{TF}}_{x}\\ \Delta LC_{x_{3}} \end{array}\right] \end{array}$$

where it is possible to use x=l or x=r to obtain forces ${\mathrm{PF}}_{l}$ and ${\mathrm{PF}}_{r}$, respectively.

## 3. Experimental Procedures

### 3.1. Subjects

Ten healthy subjects (2 women and 8 men), with ages ranging from 20 to 44 years (mean ± SD: 28.1 ± 8.2), volunteered to participate in the study.

The exclusion criteria considered were diagnoses of any disorders that might affect motor performance or the ability to respond to verbal instructions.

All the subjects declared to be in accordance with the study procedures, approved by the Ethics Committee of the University Hospital of the University of São Paulo.

### 3.2. Motor Task and Experimental Protocol

**Figure 5:**
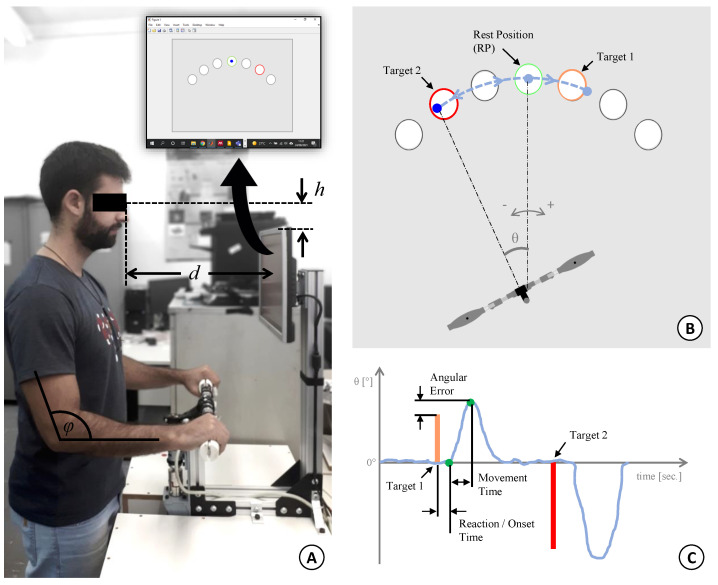
(**A**) The robotic handlebar during a healthy subject interaction. Before the experiment, the device was adjusted to each participant to respect the following ranges: −30 mm < h < 30 mm, 400 mm < d < 450 mm e $60^{\circ }<\phi <90^{\circ }$. The inset figure shows the graphical interface developed in MATLAB^®^. (**B**) Illustration of the visual interface. The blue dot cursor is controlled by the user, and it goes through the centre of each circle. The trial target is the one with a red outline. (**C**) Performance parameters: spatial error (SpTE), reaction time (RT)/onset time (OT) and movement velocity (obtained through movement time, MT).

Before starting the experiment, the volunteers stood up in front of the device, with the centre of the monitor at eye level, ensuring to reach the handlebar in a comfortable manner. To reduce the variability in positioning between participants, the height (*h*) of the eye level and the top of the monitor, the distance (*d*) from the eyes to the monitor and the elbow angle (ϕ) were kept in a narrow range, sufficient to respect the differences in biomechanic aspects among subjects (Figure 5A).

When rotating the handlebar, the subject can control a blue dot cursor and, on the gray background, seven white circles are displayed, spread within an imaginary semi-circle in different angular positions: $-45^{\circ }$, $-30^{\circ }$, $-15^{\circ }$, $0^{\circ }$ (center or rest position for the handlebar), $15^{\circ }$, $30^{\circ }$, $45^{\circ }$ (Figure 5B). All the movements start in the centre position (inside the $0^{\circ }$ circle), move to one of the targets (indicated by the red circle outline) and return to the initial position. Targets always appear in a fixed frequency of 1 Hz and the instructions were to follow them, making movements as fast as possible, without corrections.

**Figure 6:**
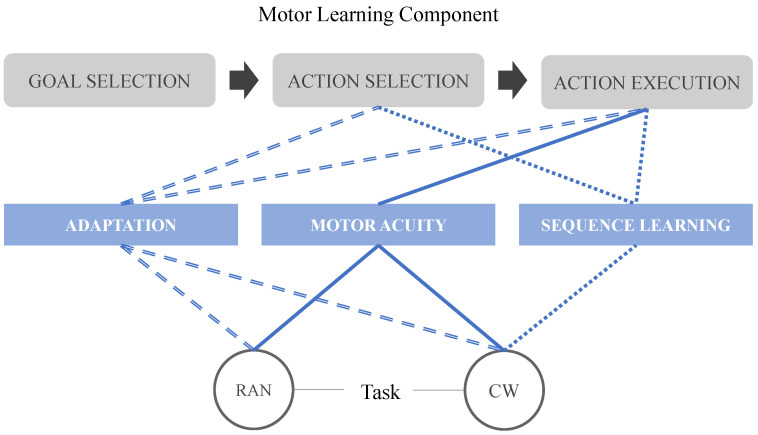
Framework for the proposed motor tasks (bottom layer) and their relationship with their respective motor learning paradigm (middle layer) and components (top layer).

The complete experimental protocol was divided into 2 sections with different motor tasks (RAN and CW) created to explore different components of the motor learning process [48]. Figure 6 shows a diagram illustrating the relationship between the proposed motor tasks and the motor components. The random (RAN) section was designed in a reaction time mode; targets were thus displayed in a pseudorandom sequence. Subjects were instructed to avoid anticipating the target but to reduce the reaction time as much as possible. The sequence (CW) section works in a timed-response mode, in which targets were shown in a clockwise (from $-45^{\circ }$ to $45^{\circ }$) order. Subjects had been previously informed about the sequence and were allowed to anticipate the targets appearance, trying to keep the synchrony.

All the sections were composed of 11 cycles of 6 targets each. In order to be familiar with the system rules and to its control dynamics, all the volunteers started by executing 10 trials (10 targets), in RAN mode, as a practice turn. Next, they all went through the RAN section, followed by a 5-minute break and, finally, the CW section. The signals produced during the practice trial were not considered during the data analysis.

Throughout the three tasks described above (including the practice trial), the system actuates to contribute with the subject’s movement, compensating for the natural friction and inertia.

### 3.3. Performance Parameters

Both kinematic and dynamic data were collected during the RAN and CW sections.

The dynamic data set comprises the force measurements recorded by all load cells, as shown in Figure 1C and calculated through Equations (8) and (10). Additionally, according to Figure 5C, three kinematic measurements were collected:

1. Spatial Error (SpTE): calculated by the difference (modulus) between the maximal angular position reached before returning to the rest position. The reference is the centre of the target;
2. Reaction Time (RT) or Onset Time (OT): it is the elapsed time from the moment the target is shown to the subject when the movement starts. It is worth noting that this measurement is positive for the RAN mode, and it represents the volunteer’s reaction time. However, during the CW mode, it is interpreted as the onset time (negative value), since the subject is always trying to anticipate the target’s appearance;
3. Movement Time (MT): defined as the elapsed time from the moment the user starts moving until the target is reached. In the following sections, the authors present these data as the movement velocity (MV), to get a normalized value based on the angular distance displaced by the user. Thus, the MV is defined as the ratio between the final angular position and the MT.

### 3.4. Data Analysis

The performance data were analysed by calculating the mean and standard error per cycle, and analysing the evolution in time. For the kinematic measurements, an algorithm (MATLAB^®^) was developed to automatically detect the moment the user started moving the handlebar, and when reaching its final position. After running the code, the detected points underwent visual inspection as a double check, correcting them whenever necessary.

In terms of statistical analysis, first all the data were evaluated in terms of normality (Shapiro–Wilk), equal variance (Brown–Forsythe) and outliers (Grubbs). Unless specified in the Results section, they all passed.

Differences among means across the cycles were assessed with repeated-measures ANOVA or Friedman (for the non-parametric case) and post hoc tests (Tukey).

For differences between groups (e.g., RAN and CW, or dominant and non-dominant hands), the *t*-test (parametric) or the Mann–Whitney test (non-parametric) was used.

Statistical significance was accepted at p=0.05, in two-tailed tests. The procedures were performed with MINITAB^®^ 19.0 (State College, PA, USA) and SIGMAPLOT^®^ 14.0 (San Jose, CA, USA); graphs were generated through MATLAB^®^ 9.6 (Natick, MA, USA).

## 4. Results

**Figure 7:**
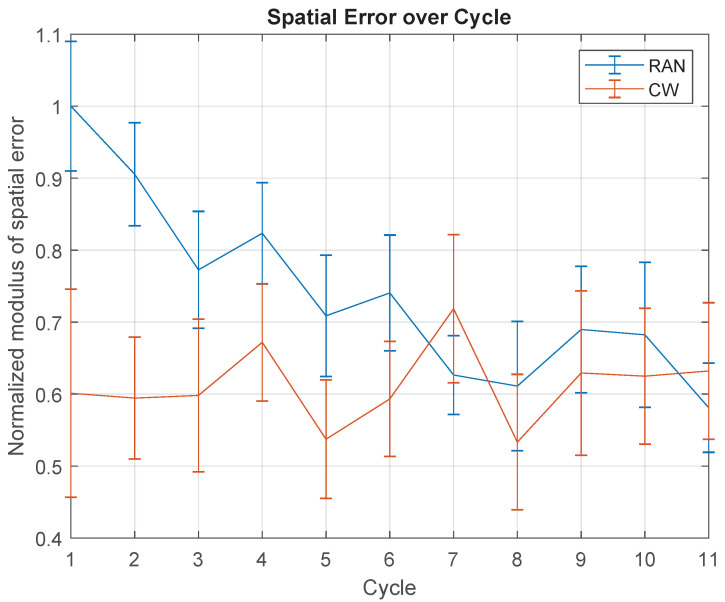
Normalised modulus of spatial error (SpTE), over cycles, for RAN (blue) and CW (red) modes. The normalisation was based on the mean SpTE obtained in the first cycle of the RAN section.

Figure 7 highlights the SpTE over cycles, for the RAN and CW modes. The mean values were normalised based on the mean SpTE obtained in the first cycle of the RAN section.

In the RAN mode, there is a gradual decrease in the average SpTE, which is related to the cycles’ evolution (F(10,90)=3.741,p≤0.000). In turn, throughout the CW section, there is no significant statistical difference of mean SpTE over time (F(10,80)=0.575,p=0.830). The RAN and CW averages can be considered indistinguishable only after the 7th cycle (Tukey test, α=0.05). Indeed, comparing RAN and CW, the mean SpTE across the first 6 cycles, there is a difference of $0.682\pm 0.291^{\circ }$ (t(8)=2.34,p=0.047), meaning participants reached a higher level of accuracy throughout the CW section when compared to RAN.

**Figure 8:**
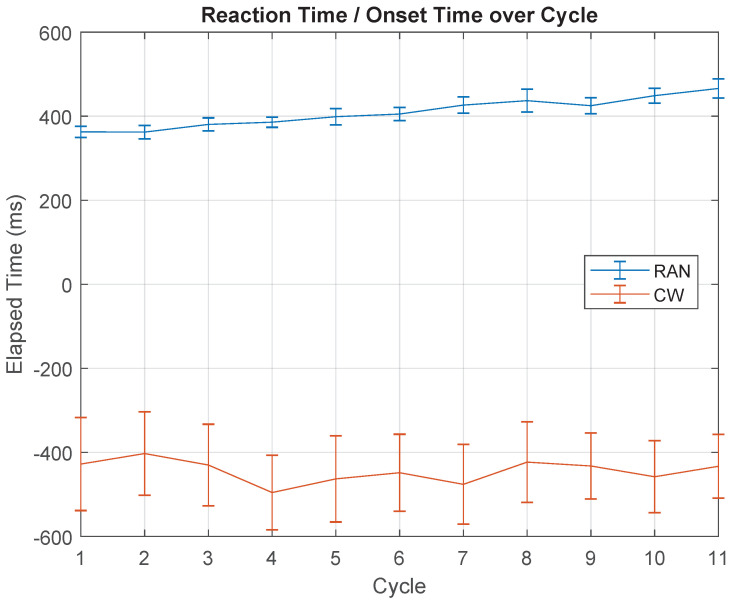
Reaction time (RT) and onset time (OT), over cycles, for RAN (blue) and CW (red) modes. RT is related to RAN and OT refers to CW. Since the user knows the position of the upcoming target, the movement always anticipates the target appearance; hence, OT is defined as a negative value.

In terms of reaction and onset time, the graph in Figure 8 shows that, in the RAN mode, the mean RT suffered a slight increase over the cycles ($\chi^{2}\left(10\right)=72.610,p\leq 0.001$, whereby one subject was removed after the outlier test: G(10)=2.47,p=0.012), whereas in CW, a constant mean OT throughout cycles was observed ($\chi^{2}\left(10\right)=5.950,p=0.820$).

**Figure 9:**
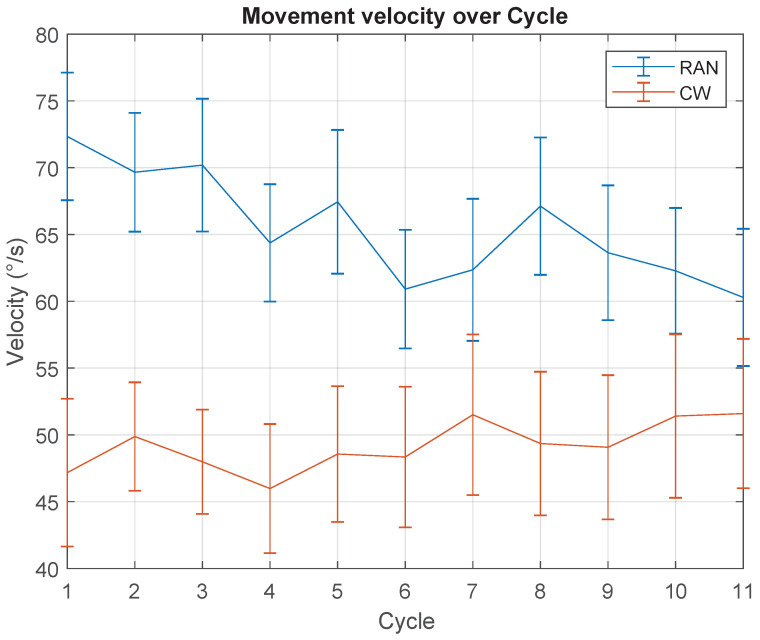
Movement velocity (MV), over cycles, for RAN (blue) and CW (red) modes. MV is obtained through the movement time, as described in Figure 5.

The movement velocity was obtained for both scenarios, RAN and CW. Figure 9 shows a decrease in mean MV over cycles, in terms of RAN mode (F(10,90)=2.476,p=0.011), and a constant trend during the CW task ($\chi^{2}\left(10\right)=8.290,p=0.601$).

**Figure 10:**
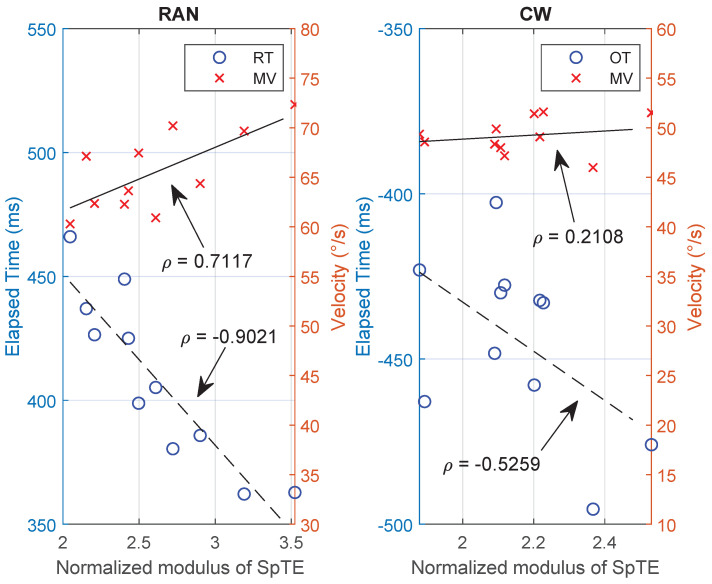
Correlation between reaction time, RT (or onset time, OT) and movement velocity (MV), with the normalised modulus of spatial error (SpTE), for both scenarios, RAN (graph on the left) and CW (graph on the right).

Based on the three graphs of the kinematic data presented above, it seems the performance parameters in RAN mode are correlated with each other, whereas no specific trend could be inferred from the CW data set. Figure 10 draws the assumed correlation for both scenarios and corroborates the first impression.

**Figure 11:**
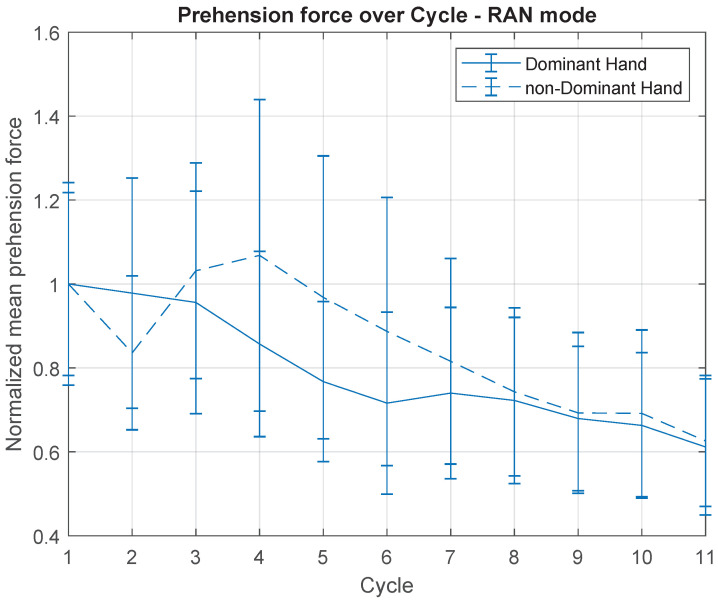
Mean prehension force, over cycles, for dominant and non-dominant hands, only for RAN task. Normalisation was carried out based on the mean value of the first cycle, for each hand.

**Figure 12:**
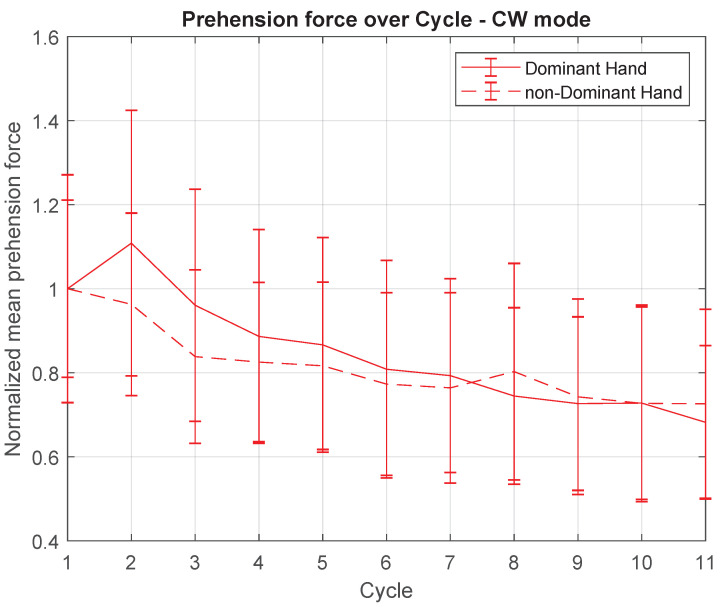
Mean prehension force, over cycles, for dominant and non-dominant hands, only for CW task. Normalisation was carried out based on the mean value of the first cycle, for each hand.

With regard to the dynamic measurements, Figure 11 and Figure 12 point out the mean prehension force of each hand (normalised by the mean value of first cycle), in RAN and CW practices, respectively.

Differently from what was observed in the kinematic data, in which only the RAN measurements seem to have a significant dynamic behaviour throughout the cycles (Figure 10), throughout the dynamic recordings (prehension force), both RAN and CW modes seem to present a similar decay across cycles. Statistical results comparing the mean force of each cycle in the RAN section were (Friedman test): dominant hand—$\chi^{2}\left(10\right)=23.4,p=0.009$, and non-dominant hand—$\chi^{2}\left(10\right)=34.51,p\leq 0.000$; and in the CW section, they were (Friedman test): dominant hand— $\chi^{2}\left(10\right)=22.24,p=0.014$, and non-dominant hand—$\chi^{2}\left(10\right)=21.85,p=0.016$. Additionally, in RAN, the overall mean prehension forces of the dominant and non-dominant hands were statistically the same (t(20)=0.89,p=0.386). In CW, the mean prehension force of the non-dominant hand was 66% of the mean value of the dominant hand (t(20)=6.58,p≤0.000).

**Figure 13:**
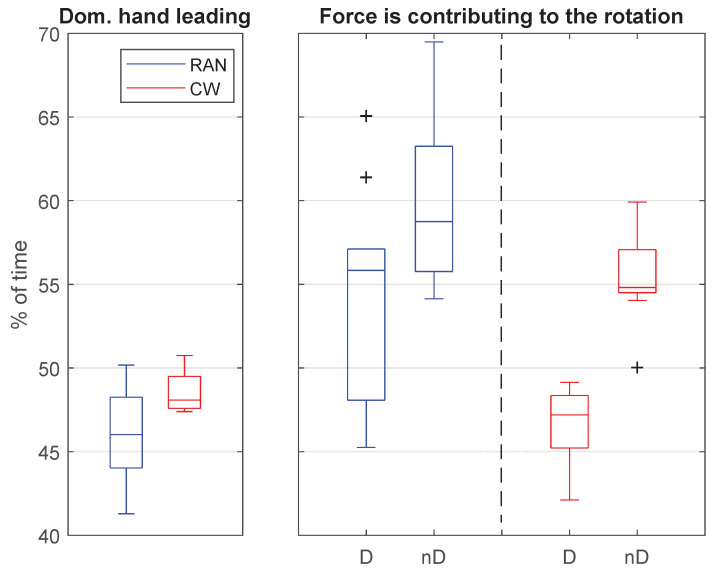
(**Left**): comparison of percentage of time the dominant hand spends leading the task, during the RAN and CW tasks. (**Right**): comparison of percentage of time in which both hands contribute to the handlebar rotation, during RAN and CW modes.

The graphs in Figure 13 were built using the forces parallel to the body transverse plane (see Figure 1).

In terms of the hand that leads the task, the graph on the left in Figure 13 shows that the dominant and non-dominant hands approximately share the task leadership evenly. Comparing RAN and CW, the first shows greater variability but lower mean value for the percentage of time leading the task (Mann–Whitney test: W=91.00,p=0.022).

The graph on the right in Figure 13 shows the percentage of time when the force of each hand is applied so as to contribute to the desired handlebar rotation. According to this definition of a contributing force, when the limb is not contributing to the desired handlebar rotation, it is acting to break the other limb force. The graph shows that, either in RAN or in CW, the non-dominant hand spends more time contributing to the desired movement than opposing it, whereas the dominant hand operates in the opposite way (RAN: difference of 6.66±6.11%, t(20)=2.55,p=0.019; CW: difference of 8.94±2.42%, t(20)=8.66,p≤0.000). Once again, the variability levels were higher in the RAN than in the CW task.

**Figure 14:**
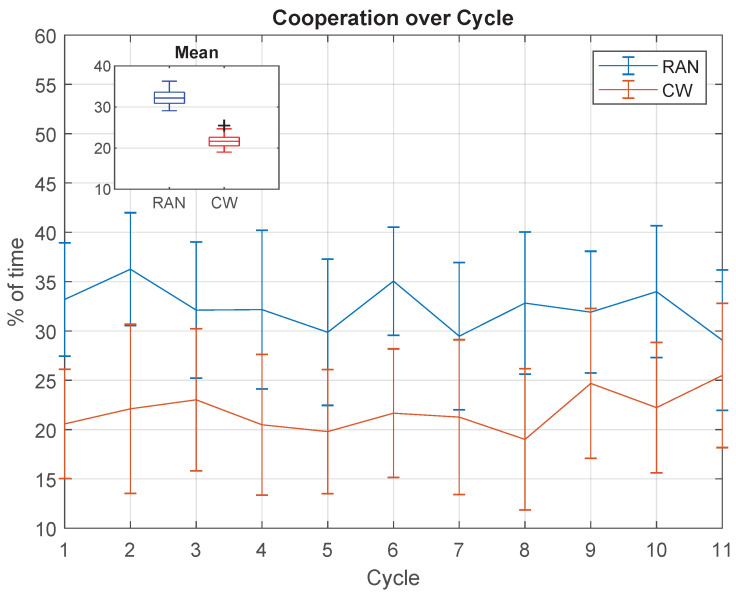
Mean percentage of time when hands cooperate, which means that both act to rotate the handlebar in the same direction (clockwise or counter-clockwise).

Finally, Figure 14 shows the percentage of time when both hands cooperate in each cycle. According to the proposed definition, hands cooperate when both act together to rotate the handlebar in the same direction (clockwise or counter-clockwise). The graph shows that during the RAN task, the hands cooperate more than in CW (difference of 10.51±3.26%, t(10)=10.69,p≤0.000), and the mean value is approximately constant over the cycle in both scenarios (RAN: $\chi^{2}\left(10\right)=5.39,p=0.863$; CW: $\chi^{2}\left(10\right)=3.81,p=0.956$).

## 5. Discussion

In this work, we presented a bimanual robotic device along with a set of motor control and learning experiments that validated this approach and paves the way for future rehabilitation devices. The assessment of the bimanual tasks allowed us to test several hypotheses about motor learning.

### 5.1. Movement Accuracy Increased with Practice Only in the Random (RAN) Task

Figure 7 shows a gradual decrease of the average SpTE, according to the progression over the proposed exercise, in RAN mode. This is usually related to the practice in the task that leads to an adaptation to the conditions of the activity and system dynamics, consequently increasing the accuracy level. In this context, during the clockwise sequence (CW) task, no adaptation was observed. A possible explanation for the distinct results in both cases is that they did not demand the same level of difficulty, being the CW scenario less challenging than the previous one. Another interpretation is that, in CW, once participants were aware of the targets sequence, they changed their motor strategy to plan and execute actions.

The latter explanation is suggested by a prior study by Ghilardi and colleagues [48], whereby a similar task was implemented, but using a unimanual device with linear displacement in multiple directions, involving healthy subjects. In terms of spatial error, the authors reported results similar to what is shown in Figure 7.

Other studies point to the same direction, demonstrating that when the targets appear in a random way and the volunteers are instructed to react to them as fast as possible (RAN mode), the motor trajectory planning is abruptly interrupted, and is thus incomplete when the subject starts the movement [49,50]. In turn, during the CW practice, the target anticipation is possible, thus increasing the time for parameter specification and also for the movement execution itself, leading to improvement in accuracy [51,52,53].

### 5.2. Reaction Time Increased with Practice Only in RAN Scenario

From the graph shown in Figure 8, there was a slight increase in RT across cycles. According to what was discussed previously, it may indicate a user’s attempt to increase the available time for parameter specification, in comparison to the truncated trajectory planning (peculiar to reaction time tasks) that participants show during the first trails of the activity.

Note that, even though all participants were instructed to react as soon as possible to the visual stimulus, the RT increased during the RAN activity. However, together with this orientation, participants received another instruction, to make fast movements without corrections, struggling to reverse the movement right at the centre of the target (minimal SpTE). Both instructions are somewhat concurrent since greater accuracy in general requires longer planing time (increasing reaction time). In this sense, the result for RAN mode, in Figure 8, reveals the implicit content of the (non-declarative) decision made by each individual, in each trial. It seems that, on average, participants opted to increase the spatial accuracy at the expense of also increasing the RT, perhaps because the time marks during the activity were not so visual and straightforward as the spatial clues were. Finally, for respecting both given instructions (repeated during the trials), volunteers implicitly optimized their available time between the appearance of two consecutive targets, spending more time planning the trajectory and the action.

### 5.3. Movement Velocity Decreased with Practice Only in the RAN Scenario

Once again, apparently against the given instructions to make fast movements, the graph in Figure 9 shows a decrease in the movement velocity over the cycles, for the RAN task. Following the same explanation, it seems the result of an implicit decision to increase accuracy, which is directly related to Fitt’s law [54] since this implies the reduction of movement velocity.

In terms of RAN mode, from what has been discussed so far, the results suggest an inverse relationship between SpTE and RT (the longer the time spent to plan the action, the greater is the accuracy reached) and a direct relationship between SpTE and MV (Fitts’ law). These correlations are clearer in the left graph of Figure 10 and they indicate adaptation in the overall performance.

The same plot, but for the CW scenario, is presented in the right graph of Figure 10. In this case, it is possible to see a low correlation between variables, which is connected to the idea discussed in the first paragraph of this discussion, suggesting that a low-level or no motor adaptation process has happened.

These results corroborate the literature, although, so far, a few or no studies have involved these kinematic analyses, elaborating on adaptation in different motor learning scenarios, with a bimanual task as we presented in this work.

### 5.4. Prehension Force Decreased with Practice, in RAN and CW Modes

Another important aspect discussed herein is the dynamic measurements the handlebar is able to record. Taking the RAN task first, Figure 11 shows the mean prehension force for dominant and non-dominant hands, for each cycle.

Nevertheless, the visual analysis of the curves in Figure 11 reveals that the mean prehension force of both hands decreased throughout the activity. This fact may be related to the motor adaptation described before, suggesting that the adaptation process led to a more natural and automatic control of the handlebar, which means a more relaxed interaction with the device (reducing co-contraction). From this perspective, the kinematic recordings of the handlebar represent an alternative roll of parameters from which a certain motor performance can be analysed. This is fundamental for understanding the importance of developing novel devices able to collect dynamic data, in addition to the kinematic, and use them to infer about the nature of the learning or adaptation process helping, for example, with the hard work of distinguishing between the implicit and explicit parcels of a certain learning process.

All the subjects received the same instructions before initiating the activities, and none of them involved the prehension force. This means that the reduction of this force during the activity represents an implicit process. Although the effects of this implicit process on the motor performance (e.g., spatial accuracy) remain to be investigated in further studies, the results in Figure 11 illustrate an important feature of the device presented.

The same interpretation can be applied to the graph in Figure 12, obtained for the CW scenario. Although the kinematic data shown in Figure 7, Figure 8 and Figure 9 did not indicate any kind of adaptation process during this task mode, after analysing from the dynamic measurements perspective, the same implicit release of the handles seemed to happen, for both hands. As mentioned before, the effects of this implicit adaptation process need to be better investigated. If not related to motor performance it can be, for example, part of another type of adaptation whose aim is to optimize energy loss, reducing the prehension force according to the practice in the task.

### 5.5. Dominant Hand Did Not Lead the Task

Results also showed that the dominant and non-dominant hands approximately share the task leadership evenly, in both scenarios, RAN and CW, which suggests no lateralization during the execution of the proposed bimanual tasks (left graph in Figure 13). However, when the forces of both hands are evaluated in terms of their contribution to the handlebar rotation, it seems the hands play different roles within the motor execution (right graph in Figure 13). Since the dominant hand spent more time working in opposition to the handlebar rotation, it can be interpreted as a breaking movement, controlling the final position. In turn, the non-dominant hand moves according to the actual rotation in more than 50% of its execution time, suggesting it is responsible for the gross displacement of the handlebar.

In terms of variability, the RAN task mean value was always more variable than the one obtained through CW. This may be linked to the adaptation process observed in the RAN task. During the execution of a novel motor task that demands adaptation to the system parameters, the subject might run different motor strategies that eventually create a scenario with variable use of the limbs, resulting in the observed greater variability.

### 5.6. Cooperation between Limbs Remained Constant over the Task

The forces parallel to the body traverse plane were also analysed according to their mutual cooperation. If the dominant and the non-dominant hands work together, reciprocally adding to the same angular movement, they are defined as cooperative forces, at that specific moment. According to the results in Figure 14, hands cooperate more throughout the RAN than in the CW mode, which can also be associated to the adaptation process more pronounced in the RAN task. Interestingly, the mean cooperation between hands remained constant over the cycles, which suggests that, in the case of RAN, this feature is not associated to the improvement in spatial error.

We believe the results discussed above are important for the comprehension of motor control and learning processes in bimanual tasks. This understanding is crucial to the effective translation from research laboratories to clinical settings. However, in future works, we also intend to investigate the use of the robotic handlebar for rehabilitation of hemiplegic patients. In this sense, usability features should be improved. For example, due to spasticity or hand weakness, some patients may have difficulty to properly grip the handles, thus the grip strength recording would be compromised. Addressing this issue, fabric straps may be used to comfortably secure the patient’s hands. Similarly, in some cases it may be necessary to use an external elbow/arm support system. Moreover, the wood desk where the apparatus is mounted must be modified in order to accommodate users in sitting position, which is particularly important for wheelchair users. Additionally, since motivation and engagement is a key factor of successful rehabilitation protocols, ludic, graphical and interactive games should be developed and incorporated to the system.

## 6. Conclusions

Motor control and motor learning research is often supported by instrumented and actuated devices (e.g., robots), although they usually perform unilateral tasks.

From a neuronal perspective, the control of both arms is not equivalent to the superposition of controlling each limb separately. Thus, the understanding of motor control and learning mechanisms in bilateral manipulation is crucial to develop rehabilitation devices that effectively address interlimb coordination function recovery.

Among the devices that explore the bilateral motor control and learning processes, they mostly focus on kinematic measurements, such as movement velocity, while neglecting dynamic data, such as prehension force, which is important to understand the nature of motor learning mechanisms.

In this context, this work presented the construction, modelling and validation of a robotic device designed to implement different motor learning experimental paradigms. It emulates a bicycle handlebar, thus requiring a bilateral task, with rotational movements and a coordinated sequence of joint sub-movements. The robotic handlebar is compact and portable, and can register both position and forces. Moreover, the system is able to generate force fields, thus suitable for implementing motor learning paradigms and rehabilitation protocols. The impedance control system also promotes a safer environment for human interaction.

A pilot test with ten healthy subjects performing two tasks, RAN (reaction time task) and CW (timed-response paradigm), was presented. The results indicated that movement velocity decreased with practice along with the increase in reaction time, indicating a possible increase of the task planning time. Moreover, the prehension force decreased with practice and, more interestingly, the dominant limb did not lead the task underscoring the role of bimanual coordination in handlebar control. While this might be surprising, it may be related to the resultant force. In this respect, it is also possible that the non-dominant arm applies a constant force to facilitate the task from an impedance control perspective.

Future research includes the investigation of hand dominance influence on the task as a function of the lateralization degree. Furthermore, we expect to explore the use of the robotic handlebar as a rehabilitation tool in clinics and research institutions. Thus, we plan to conduct pilot experiments with stroke survivors as well as a viability study to evaluate the production and commercialization costs of the device.

## Figures and Tables

**Table 1:** Characteristics of the robotic handlebar.

L	m	*l*	$l_{c}$
0.555 m	0.98 kg	0.12 m	0.08 m
